# In-/off-label use of biologic therapy in systemic lupus erythematosus

**DOI:** 10.1186/1741-7015-12-30

**Published:** 2014-02-17

**Authors:** Mariele Gatto, Emese Kiss, Yaakov Naparstek, Andrea Doria

**Affiliations:** 1 Division of Rheumatology, University of Padova, Via Giustiniani, 2, Padova 35128, Italy; 2 Department of Clinical Immunology, Adult- and Paediatric Rheumatology, National Institute of Rheumatology and Physiotherapy, Budapest, Hungary; 3 Rheumatology Division, 3rd Department of Internal Medicine, Semmelweis University, Budapest, Hungary; 4 Senior Deputy Director General for Research and Academic Affairs, Hadassah Medical Organization, Hadassah Hebrew University Medical Center, Jerusalem 12000, Israel

**Keywords:** Systemic lupus erythematosus, Biologic therapy, Randomized controlled trials, Belimumab, Anti-B cell therapies

## Abstract

Current therapies for systemic lupus erythematosus (SLE) include corticosteroids as a persistent mainstay and traditional immunosuppressants which are given according to disease severity, organ involvement and patient status. No treatment entails certain efficacy devoid of mild-to-moderate adverse effects. Nowadays, novel therapies are being developed aiming to target specific molecules involved in SLE development and progression which show variable effectiveness and safety. Biologic agents considered for SLE comprise monoclonal antibodies (chimeric, humanized or fully human) as well as fusion molecules or antibody fragments mostly consisting of B cell-targeted therapies beside anti-cytokines as well as T cell-targeted therapies. Encouraging evidence on biologics is mostly provided by case series or uncontrolled studies; conversely, larger randomized controlled clinical trials have frequently missed their primary endpoints with the exception of BLISS-52 and BLISS-76 trials. Actually, apart from belimumab, biologics are employed in clinical practice as off-label treatments for lupus and results are often promising, depending on specific SLE features, dose regimens and individual responsiveness.

## Background

In recent times, hope was raised in systemic lupus erythematosus (SLE) for biologic therapies that are capable of specific molecular targeting at distinct key points. Novel therapies tested in SLE comprise anti-cytokines antibodies (for example, anti-interleukin(IL)-6), anti-B cell therapies targeting either B cell surface antigens (anti-CD20, anti-CD22) or B cell survival factors (for example, anti-BLyS/APRIL monoclonal antibodies), as well as drugs intervening in B-T cell co-stimulation (CTLA4-Ig) [1-12]. It has to be noted that with the exception of belimumab most of these drugs are far from being dispensed in clinical practice [1,13], and some will never become available since randomized controlled trials (RCTs) have missed their primary endpoints despite promising preliminary results. Hence, apart from belimumab, biological treatment of SLE still remains an “off-label matter”, raising conflicting opinions on reasons for RCTs’ failure and biologics’ suitability in lupus management. The aim of our commentary is to provide an overview of candidate biological therapies for SLE, in light of studies which challenge or support their applicability in clinical practice.

### B-cell targeted therapies

B-cell targeted therapies represent the bulk of candidate biologic medications in SLE, with the key role consistently played by B cells in disease pathogenesis and progression [14] (Table 1).

**Table 1:** **Biologic therapies proposed for SLE**^**a**^

	**Drug**	**Type of molecule**	**RCT phase**	**Pros for use in SLE**	**Cons for use in SLE**	**References**
Anti-B cell therapies	Rituximab	Chimeric anti-CD20 mAb	Phase III	• Significantly decreased anti-dsDNA antibody titers and increased complement levels vs. placebo. Significantly decreased lupus non-renal flares in Hispanic and African American patients	• Did not significantly improve lupus outcome in either renal or non-renal SLE in large cohorts of SLE patients	[2,3]
	Ocrelizumab	Humanized anti-CD20 mAb	Phase III	• Significantly increased complement levels and decreased anti-dsDNA titers through week 48 vs. placebo	• No significant improvement in renal outcome	[15]
				• Induced numerically (non-statistically) significant greater renal response vs. placebo	• Serious infections in treated patients vs. placebo when added to MMF	
	Epratuzumab	Humanized anti-CD22 mAb	Phase IIb	• Provided clinical improvement in patients receiving ≥2,400 mg cumulative dose	• No significant BILAG improvement after 12 weeks treatment vs. placebo	[5]
				• Steroid-sparing effect		
	Belimumab	Fully human anti-BlyS mAb	Phase III	• Significant improvement in moderate-persistent active SLE outcome and decreased flare rates in phase III RCTs (met primary endpoints)	• No BILAG assessment	[8,9]
					No data on CNS or severe renal involvement	
				• Significant decrease in anti-dsDNA antibody titers	• No advantages by treatment continuation through week 76 (but in *post-hoc* analysis)	
				• Steroid-sparing effect		
	Atacicept	Soluble fully human recombinant anti-APRIL fusion protein	Phase II	• Not available^b^	• Serious infections and decreased immunoglobulin levels in patients receiving MMF or corticosteroids prior to atacicept	[6]
Anti-co-stimulatory molecules	Abatacept	CTLA4-Ig fusion protein	Phase IIb	• Reduced BILAG A polyarthritis flares in a phase IIb RCT	• Did not reduce overall disease flares vs. placebo in either renal or non-renal SLE	[10]
	IDEC-131	Humanized anti-CD40 ligand mAb	Phase II	• Ameliorated SLEDAI in a phase II RCT	• No significant superiority to placebo	[11,12]
					• Increased risk of thromboembolic events	
Anti-cytokines therapies	Infliximab	Chimeric anti-TNF soluble mAb	No RCT performed	• Effective on refractory arthritis, nephritis and skin lesions in open-label studies	• Severe adverse events following treatment, for example, thrombosis, infections	[16,17]
				• Reduction in SLEDAI and SLICC-DI in pilot studies after short-term induction treatment	• Induction of pathological/pathogenetic autoantibodies (anti-dsDNA, anti-phospholipid antibodies)	
					• Increase in IFNα levels following protracted administration	
	Tocilizumab	Humanized IgG1 anti-IL6R mAb	Phase I	• Significantly reduced SELENA-SLEDAI score (main improvement on arthritis)	• Neutropenia and serious infections	[18]
				• Significantly reduced IgG anti-dsDNA antibody levels	• No data on severe SLE	
	Anakinra	Non glycosylated IL1Ra	No RCT performed	• Improved arthritis in an open-label trial	• No long-lasting effect	[4]
					• No extensive data are available due to very low patients number	
	Sifalimumab	Human mAb blocking multiple IFNα subtypes	Phase I	• Significantly reduced the rate of disease flares vs. placebo	• No data on severe SLE	[19]
				• Lower request of immunosupressor vs. placebo		

^a^Only biologic therapies for which published clinical studies are currently available are listed.

^b^the study was prematurely terminated and no efficacy measures were undertaken.

anti-dsDNA, anti-double stranded DNA; APRIL, A PRoliferation Inducing Ligand; BILAG, British Isles Lupus Assessment Group; BLyS, B Lymphocyte Stimulator; CNS, central nervous system; IFNα, interferon alpha; IgG, class G immunoglobulin; IL1Ra, interleukin 1 receptor antagonist; IL6R, interleukin-6 receptor; mAb, monoclonal antibody; MMF, mycophenolate mofetil; RCT, randomized controlled trial; SLE, systemic lupus erythematosus; SLEDAI, Systemic Lupus Erythematosus Disease Activity Index; SLICC-DI, Systemic Lupus International Collaborating Clinics group Damage Index; TNFα, tumor necrosis factor alpha.

#### ***Rituximab and anti-CD20***

CD20 is a B lymphocyte restricted cell surface molecule that is expressed from pre-B to memory B cells, but is not found in stem cells and plasma cells. Rituximab (RTX) is a chimeric antibody against the CD20 molecule which was tested in two RCTs [2,3]. The EXPLORER (Exploratory Phase II/III SLE Evaluation of Rituximab) trial was aimed to assess RTX efficacy and safety in moderate-to-severe active non-renal SLE. RTX depleted B cells but did not significantly reduce anti-DNA levels. Unfortunately, no differences in the British Isles Lupus Assessment Group (BILAG) score and flare-free ratio were observed. The Lupus Nephritis Assessment with Rituximab (LUNAR) study involved patients with proliferative lupus nephritis (LN) [5]. The primary outcome was the proportion of patients with complete or partial remission of LN at 12 months, which was not attained; two deaths (sepsis and pneumonitis) occurred in the RTX group. Evaluation of Ocrelizumab (humanized anti-CD20 monoclonal antibody, mAb) in two phase III RCTs - the BEGIN (A Study to Evaluate Two Doses of Ocrelizumab in Patients With Active Systemic Lupus Erythematosus) and the BELONG (A Study to Evaluate Ocrelizumab in Patients With Nephritis Due to Systemic Lupus Erythematosus) very similar to the EXPLORER and LUNAR - was stopped prematurely due to an increase in infections in the active arm of the studies [15].

Different facts can explain the failure of these phase III RCTs testing CD-20 depleting agents in SLE, for example, the low number of patients, highly variable background of immunosuppressive therapy and high dose steroids, relatively short follow-up period, non-adequate outcome measures and, importantly, extreme endpoint stringency (no BILAG A or B, and complete renal response)*.* However, partial favorable reports for RTX were documented in that the EXPLORER trial showed a higher percentage of complete or partial response at week 52 in African American and Hispanic patients. Statistically significant improvements in serum complement (C3 and C4) levels and decreases in anti-dsDNA antibody levels were observed among RTX-treated patients both in the EXPLORER and in the LUNAR trials [2,3].

Moreover, open and uncontrolled clinical studies with RTX as well as results of French, UK and other European registries indicate promising outcomes [20-22]. Prospective and retrospective studies, as well as case series and single case reports, showed 300 patients with refractory LN being treated with RTX at different dosing regimens and analysis revealed complete or partial response to RTX in approximately two thirds of patients [23]. Notably, longitudinal observations on 50 patients with proliferative LN showed promising results following treatment with RTX 1 g fortnight and pulse methylprednisolone but no oral steroids in the follow-up [24]. In this study, the majority of patients were kept on partial or complete renal remission with only mycophenolate mofetil after RTX induction. However, the definition of renal remission given in this study may be objectionable and systemic as well as renal flares occurred in a relevant percentage of patients within one year.

Nevertheless, in light of “experience-based” medicine, RTX use is included in the European League Against Rheumatism (EULAR) recommendations for refractory LN [25].

#### ***Anti-CD22 and Epratuzumab***

Epratuzumab is a humanized monoclonal antibody which activates the CD22 regulatory pathway on B cells [26]. It was tested in a phase IIb trial [5], where it caused a modest decrease of about 30% in B cells, without a change in immunoglobulin levels. The proportion of responders was higher in all epratuzumab groups than in placebo, but overall treatment effect was not statistically significant [5]. Two international RCTs (Alleviate Lupus Affliction with Epratuzumab and Validate its Autoimmune Safety and Efficacy (ALLEVIATE) 1 and 2) evaluating epratuzumab in patients with moderately to severe active SLE were discontinued prematurely because of interruption in the drug supply [27]. Despite that, 90 patients with moderate-to-severe SLE flares responded well to epratuzumab, as it was indicated by improvements in BILAG scores and steroid sparing effects. Epratuzumab was well tolerated and significantly improved health-related quality of life. Two phase III RCTs are currently ongoing (NCT01261793; NCT01262365) [5].

#### ***BAFF/BLyS and APRIL system and belimumab***

Targeting B-cell activating factor (BAFF)/B lymphocyte stimulator (BLyS) and a proliferation inducing ligand (APRIL) seems to be a suitable option in lupus. Preclinical data from experimental models and human observations confirmed high BLyS concentrations in lupus prone mice and approximately half of human lupus patients; BLyS antagonism ameliorated disease symptoms and survival in animal models [28]. When comparing RTX and belimumab, one can observe that terminally differentiated B cells are under BLyS influence but do not express the CD20 molecule, thus belimumab is expected to act faster, providing a milder immunesuppression, which is favorable by safety issues.

Up to the present, two phase III RCTs have been performed with belimumab [8,9]. The arrangement and protocols were similar; therefore combined results have also been analyzed. Antinuclear antibodies (ANA) or anti-ds(double stranded)DNA positive patients with moderately active lupus were targeted. Pooled data from the two trials indicated that a significantly higher number of patients achieved good response with both 1 and 10 mg/kg belimumab at week 52 as compared to the placebo group [8]. Furthermore, the duration of response was longer in the treated groups and the higher the belimumab dosage, the more likely corticosteroids could be reduced. In this treatment arm, patients flared less frequently as indicated by the SLE flare index (SFI) and also by flare rates at week 52. Actually, however, the second phase III study, BLISS 76, could not prove a positive sustained effect after 76 weeks [9] and it has to be noted that these studies did not recruit patients with severe LN or central nervous system (CNS) involvement. Hence, many aspects in the use of belimumab remain to be clarified in real-life SLE [29], including the clinical relevance of the differences in comparison to the placebo arm; the impact of the relatively short term effect; the effect of belimumab in treatment of refractory severe disease, including renal and CNS lupus; and finally, the balance between clinical benefit and cost.

Besides belimumab, sub-cutaneous drugs blocking soluble or receptor-bound BLyS are currently being tested in phase III RCTs [1], which in case of efficacy would provide a greater advantage in terms of administration route (subcutaneous vs. intravenous) and the patients’ comfort and compliance.

A second drug aimed at suppressing the stimulation of B cells was atacicept, a chimeric molecule against APRIL. The phase II study of atacicept in patients with LN was, however, terminated after recruiting six patients because of an unexpected decrease in immunoglobulin levels and an increased number of infections [6].

### T–cell targeted therapies

B and T lymphocytes and antigen presenting cells (APCs), may communicate directly with each other. CTLA4-Ig (abatacept) is a fusion protein that blocks the interaction within the CD86/80-CD28 stimulatory molecular pair, inhibiting the interaction between APCs and T cells, cytokine production and subsequent stimulation of B cells. Abatacept was tested in two large double-blind RCTs in non-renal lupus and in patients with LN which did not meet their primary endpoints in prevention of disease flares [10], despite studies on animal models providing beneficial effects [30]. Nevertheless, *post-hoc* analysis revealed less frequent flares in treated group as compared to the placebo [31], suggesting definitions of disease flare might have blurred clinically meaningful results albeit not statistically significant. Particularly, abatacept seems to have prevented BILAG A polyarthritis flares in patients displaying non-life-threatening SLE manifestations [10].

### Other treatment options: TNF-blocking therapies

The role of tumor necrosis factor alpha (TNFα) and its inhibition in lupus is debated. High levels of TNFα were demonstrated in human tissue samples [32] and the serum of SLE patients, where they correlated with disease activity; mouse models were likewise shown to reflect the same picture [33]. To date, open-label experience on a small patient series displaying refractory lupus manifestations brought to light some positive results. Refractory LN, skin lesions, hemophagocytic syndrome and arthritis showed beneficial effects following TNFα-depleting therapies [34,35]. Moreover, some pilot studies [17,34] demonstrated an improvement in Systemic Lupus Erythematosus Disease Activity Index (SLEDAI) and Systemic Lupus International Collaborating Clinics (SLICC) index upon TNFα depletion, suggesting TNFα-blockers could be taken into account as short-term induction therapy when dealing with refractory SLE. It is worth noting that no large controlled studies are yet available on anti-TNFα depletion in human SLE, therefore, no certain inference can be made.

Some other cytokines (IL-6, interferon alpha (IFNα)) are being explored as putative future therapeutic targets. Tocilizumab (anti-IL6R monoclonal antibody) use in a phase I trial seemed to decrease disease activity [18]. Likewise, interference with IFNα signaling might dampen DC and autoreactive B cells activation [19]. However, the data set is still very limited and no conclusions can be drawn on the disease course.

## Discussion and conclusions

To date, the only RCTs that succeeded and achieved their primary endpoints were BLISS-52 and BLISS-76, leading to approval of belimumab for mild-to-moderate SLE. Belimumab is the first drug dedicated to SLE 50 years after corticosteroids and antimalarials, meaning no other firm evidence could be drawn so far for any other treatment ranging from traditional immunosuppressants to new biologic drugs. This is probably due to the great heterogeneity of the disease [35], which makes it unlikely that one treatment may be suitable for all patients. These aspects should be considered when turning to RCTs, since generalization of results - either promising or disappointing - is a thorny matter in SLE [36]. Ironically, most of the target populations enrolled in RCTs are on the one hand too small to represent the entire disease variability and especially most severe lupus manifestations, and on the other hand they are not small enough to focus on benefits which may be gained by discrete patient subgroups, though not by the general population. Consistently, patients belonging to African American or Hispanic ethnicity showed positive results in RCTs which had globally failed [23], suggesting study designs are sometimes objectionable in regard to outcome measures, background medications (for example, high dose corticosteroids overwhelming any positive effect of the new drug) or shortness of follow-up period [1,23]. Moreover, since severely affected patients (for example, CNS involvement, refractory LN) are frequently excluded from RCTs, information on an emerging drug in the case of life-threatening manifestations is lacking [1]. Though going back to non-controlled trials or basing conclusions on *post-hoc* analysis would not be scientifically acceptable, endpoint definitions should reasonably correlate with clinical practice in order to provide useful indications and limit the need for off-label use of new drugs. Indeed, excessive outcome stringency (for example, no BILAG B allowed, complete renal response required) is hardly ever met in lupus, whereas partial remission or improvement in organ-specific (non-generalized) alterations is far more frequent and acceptable, yet not optimal [37]. These considerations aside, it also has to be kept in mind that compelling evidence may diverge from intuitive premises, preclinical studies and open-label trials because of the true limited efficacy of a developing drug or methodological mistakes which have to be carefully considered.

Deeper knowledge of the underlying mechanisms of lupus [35] will hopefully lead to generation of more effective targeted therapies which would both affect disease course and minimize treatment-related adverse events; nevertheless, a detailed picture of patient status [38] will always be required for the most suitable therapeutic approach to be tailored.

## Competing interests

The authors declare that they have no competing interest.

## Authors’ contributions

EK, YN, AD and MG collected data on studies and wrote different sections of the manuscript. MG merged all text and AD revised the paper. All authors read and approved the final manuscript.
